# Should the FDA's criteria for the clinical efficacy of cachexia drugs be changed? Is Ostarine safe and effective?

**DOI:** 10.1002/jcsm.12695

**Published:** 2021-03-23

**Authors:** Charles Paul Lambert

**Affiliations:** ^1^ University of California San Diego Extended Studies La Jolla CA USA

Physical function is a complex voluntary activity for humans. This involves the integration of information from cognition, the central nervous system, the peripheral nervous system, and the skeletal muscle participating in the movement.^1^ To move, i.e. physical function, requires the idea to move, an impulse from cerebral cortex which goes through the central nervous system, down the spinal cord to the motor end plate on skeletal muscle, and then skeletal is then activated to move.^1^ As one can see, there is a considerable interaction between skeletal muscle and the nervous system. The idea that anabolic agents can improve function in cachexia (skeletal muscle wasting) without ‘training of the nervous system’ is somewhat uninformed. The endpoints required for FDA approval are based on physical function while the drugs used to treat cachexia would only be expected to increase muscle mass and have no effect on the nervous system. This is the case if physical exercise is not included in combination with the drug in question. To involve the nervous system, which is almost always essential for improved physical function, some sort of exercise should be combined with the drug in question to actually improve physical function. To my knowledge, there is no evidence that non‐steroidal selective androgen receptor modulators (SARMS) affect the nervous system as they are specific to skeletal muscle. Simply adding muscle mass without training the neuromuscular system would appear to be less efficacious than combining the two interventions (SARMS and exercise).

Resistance exercise training (weight training) increases muscle strength in the first ~4–6 weeks without notable increases in muscle mass.^1^ Therefore, it is believed that it is almost all adaptations are nervous system adaptations during this first portion of resistance training adaptations. After 4–6 weeks, muscle mass/muscle size improvements are notable and continue for some time if the resistance training is progressive, with regard to added resistance (weight lifted). This increase in muscle mass may happen for years. Therefore, it would appear that there are potentially two ways to optimize drug efficacy for FDA approval: (1) require exercise training, of some type, combined with the drug in question during Phase 2 and/or 3 clinical trials and (2) remove the physical function improvement end point from FDA approval criteria of cachexia drugs as the drugs themselves have little effect on nervous system function (and likely whole body physical function) but profound effects on skeletal muscle hypertrophy.

## Ostarine (MK‐2844 and GTx‐024)

The good news is: it appears that the new drugs produced to increase muscle mass (SARMS), do just that. Additionally, their efficacy is also accompanied by a safer side effect profile than their wholly androgenic counterpart testosterone. That is testosterone is not only anabolic but has secondary male related side effects. For example, testosterone increases prostate growth and may cause prostate cancer in some patients. These new compounds are called non‐steroidal selective androgen receptor modulators (SARMS). The most notable SARM is Ostarine produced by GTx laboratories (Memphis, TN). It was shown to add statistically significant amounts of lean body mass in Phase II clinical trials; however, no improvement in physical function was observed in the study of Dobs *et al*.^2^ Dalton *et al*.,^3^ however, reported improvements in lean body mass of 1.2 kg and statistically significant improvements in physical function as stair climbing time and stair climbing power. It is unknown how much practice on the stair climbing test was utilized in this patient population. Crawford *et al*.^4^ in an article describing a proposed Phase III clinical trial, reported that, for FDA approval, responders had to see a greater or equal to 10% increase in Physical Function with Ostarine administration. To my knowledge since there is 150 patients in two groups, we are still awaiting the results of that study. In the study of Dobs *et al*.,^2^ there were adverse reactions; however, there were actually three more adverse reactions in the placebo group than the Ostarine group. Thus, adverse reactions cannot be attributable to the Ostarine, in this study. Advantages with the addition of lean body mass/muscle mass in cancer patients are longer survival and likely greater quality of life as cachexia, by definition, results in muscle mass loss and fat loss. What the ultimate goal should with these agents is that cancer survival time and quality of life was greater. In theory, the addition of muscle mass would ‘buy more time’ for tumour intensive therapies (e.g. immunotherapy, radiation, and surgery; chemotherapy may be contraindicated because of its effects on protein synthesis potentially of skeletal muscle and of RNA synthesis or transcription) to stop the cancer before the cachexia or muscle wasting results in mortality from, literally, wasting away. It has been reported that cancer cachexia or muscle wasting, can result in an 80% of mortality rate in cancer patients.^5^ So clearly, there is a role for increasing muscle mass on increasing survival times and potentially ‘buying more time’ to cure the cancer. It is beyond me why an increase in function is necessary unless you were going to try to enhance immune system function with regular physical exercise.^6^ Regular physical exercise of the aerobic and/or resistance type will indeed boost immunity in relatively healthy volunteers!^6^ If improved physical function is the goal, then these anabolic agents (i.e. Ostarine) should almost always be combined with regular physical exercise.

Lambert and Evans^7^ reported gains in muscle strength on the order of 50% or more from ~3 months of resistance exercise training in the elderly (at least 10 studies/review articles). Increases in muscle strength are clearly a precursor to improved physical function whereas simply adding muscle mass is not. Fiatarone *et al*.^8^ reported that 10 weeks of resistance training in 100 men and women mean age 87.4 resulted in a 28.4% increase in stair climbing power compared to no‐exercise training. Stair climbing power is a measure of physical function. Thus, in their group of very elderly individuals, strength training increased physical function with no other anabolic intervention.

To reiterate, (1) physical function would appear to be of little importance in cancer patients unless you were to attempt to improve immune function through chronic (months) exercise training after assessment and improvement of physical function. (2) Functional benefits can be seen with resistance training alone. (3) Drugs that increase muscle mass likely will improve (prolong) survival times and increase the amount of time (days, weeks, months, years) for traditional cancer therapeutics to work. (4) These drugs *should not* be evaluated on their ability to improve function but should be evaluated on their ability to *safely* increase lean body mass/muscle mass. The good news is that Ostarine and drugs like it appear to safely and effectively increase muscle mass at least through Phase II clinical trials.^2^, ^8^ To the best of my knowledge, we are awaiting the results of more expansive Phase III clinical trials.

## Conflict of interest

None declared.
